# Cerebrospinal Fluid Levels of Chromogranin A in Parkinson’s Disease and Multiple System Atrophy

**DOI:** 10.3390/brainsci11020141

**Published:** 2021-01-22

**Authors:** Michaela Kaiserova, Monika Chudackova, Katerina Mensikova, Miroslav Vastik, Sandra Kurcova, Hana Prikrylova Vranova, David Stejskal, Petr Kanovsky

**Affiliations:** 1 Department of Neurology, University Hospital Olomouc, I.P. Pavlova 6, 77900 Olomouc, Czech Republic; katerina.mensikova@fnol.cz (K.M.); miroslav.vastik@fnol.cz (M.V.); sandra.kurcova@fnol.cz (S.K.); petr.kanovsky@fnol.cz (P.K.); 2 Department of Neurology, Faculty of Medicine and Dentistry, Palacky University and University Hospital, Olomouc, I.P. Pavlova 6, 77900 Olomouc, Czech Republic; monika.chudackova@fnol.cz; 3 Neurology Outpatient Clinic “St. Moritz”, 77900 Olomouc, Czech Republic; hanna.prikrylova@seznam.cz; 4 Institute of Biomedical Sciences, Faculty of Medicine, Ostrava University, 70103 Ostrava, Czech Republic; david.stejskal@smn.agel.cz; 5 Institute of Laboratory Diagnostics, University Hospital Ostrava, 70103 Ostrava, Czech Republic

**Keywords:** Parkinson’s disease, multiple system atrophy, cerebrospinal fluid, chromogranin A

## Abstract

Background: Chromogranin A (CgA) and other peptides from the chromogranin–secretogranin family have been recently studied as potential biomarkers of various neurodegenerative diseases, including Parkinson’s disease (PD). Methods: We measured CgA in the cerebrospinal fluid (CSF) of 119 PD patients, 18 multiple system atrophy (MSA) patients, and 31 age-matched controls. We also correlated the values with disease duration and levodopa dose equivalent. Results: In the PD patients, CSF CgA tended to be lower than the control group (median 124.5 vs. 185.2 µg/L; *p* = 0.057); however, the results did not reach statistical significance. CSF CgA levels in MSA were significantly lower compared to the control group (median 104.4 vs. 185.2; *p* = 0.014). There was no significant difference in CSF CgA between PD and MSA patients (*p* = 0.372). There was no association between CSF CgA and disease duration or levodopa dose equivalent in PD or in MSA. Conclusions: We observed a tendency toward lower CSF CgA levels in both PD and MSA compared to the control group; however, the difference reached statistical significance only in MSA. Based on these results, CgA may have potential as a biomarker in PD and MSA, but further studies on larger numbers of patients are needed to draw conclusions.

## 1. Introduction

Chromogranin A (CgA) is an acidic soluble protein and a member of chromogranin–secretogranin family, stored and co-released from vesicles together with catecholamines. This protein is present in both the peripheral and the central nervous system [1]. CgA is known as a marker of neuroendocrine tumors [2], but it is also studied as a potential marker of other diseases, including essential hypertension [3] and various neurological diseases such as Alzheimer’s disease [4] and amyotrophic lateral sclerosis [5]. Recent studies show that CgA levels may also be altered in patients with Parkinson’s disease (PD), in both serum and cerebrospinal fluid (CSF) [6,7,8,9,10]. In the patients with early and advanced PD lower levels of CSF CgA have been found [7,8,10]. Rotunno et al. measured various proteins from the granin family in the CSF of PD patients as potential biomarkers; CgA was found to be decreased [11]. Studies concerning serum CgA in PD produced contradictory results. Xu et al. found increased serum CgA levels in PD compared to a control group, and the concentration increased from the early to later disease stage [9]. Gmitterova et al. found lower levels of CgA in serum in PD [6].

In our previous work, we found lower levels of CSF CgA in patients with early-stage PD [7,8]. CSF CgA levels were lower in PD patients with orthostatic hypotension (OH) compared to patients without OH [7]. The limitation of these studies was the small sample of patients. In the present study, we extended the sample of PD patients in whom we measured CSF CgA. We also measured CSF CgA in multiple system atrophy (MSA) patients, as MSA is characterized by severe orthostatic hypotension.

## 2. Materials and Methods

### 2.1. Patients and Methods

The study protocol, including CSF examination, was approved by the Ethics Committee of Palacky University in Olomouc; all participants gave their informed consent prior to their recruitment, and all procedures were performed in accordance with relevant guidelines and regulations.

### 2.2. Patients and Controls

Patients with PD, MSA, and a control group were included in the study. Diagnosis of PD was established according to the UK Parkinson’s Disease Society Brain Bank clinical diagnostic criteria [12]; the diagnosis of MSA was established according to the Gilman criteria [13]. In all patients and controls, lumbar puncture was performed, and CSF CgA measured. Control subjects underwent CSF examination due to lower back pain or a tension-type headache.

### 2.3. CSF Sampling Technique and Chromogranin A

The CSF samples were obtained by lumbar puncture, with the subjects seated. During each puncture, a total of 10 mL of CSF was collected in a sterile test tube. Subsequently, the CSF was morphologically assessed and centrifuged (10 min at 1100× *g* and 4 °C). Each sample was frozen to −15 °C, and the concentration of CgA was measured with the Biorobot DSX analyzer (Dynex Technologies Inc., 14340 Sullyfield Circle, Chantilly, VA, USA) in all subjects within 10 weeks of freezing. The duration of storage was the same for PD and MSA patients and for control subjects.

A sandwich ELISA was evaluated for the quantitative determination of CSF CgA (Biovendor, Brno, Czech Republic). To validate the reliability of the assay, precision and accuracy were tested. To analyze the spiking recovery, human plasma and CSF samples from three subjects were spiked with increasing amounts of recombinant protein and assayed. The mean recovery was 99.8%. The intra-assay coefficient of variation (CV) was 6%, and the inter-assay CV was always 12%. A recombinant CgA provided by Biovendor was used as the standard. The sampling technique and CgA analysis were the same as in our previous study [7].

### 2.4. Statistical Analysis

Statistical analysis was performed using IBM SPSS Statistics for Windows, Version 22.0 (Armonk, NY, USA: IBM Corp.). The Mann–Whitney U test was used to compare differences between patients and controls, as this is a nonparametric test comparing outcomes between two independent groups. The relationships between CSF CgA and selected quantitative parameters (disease duration, age, levodopa dose) were assessed using the Spearman’s correlation—a nonparametric test that measures the strength of association between two variables. Tests were performed at the significance level of 0.05.

## 3. Results

### 3.1. Patients’ Characteristics

The study included 119 patients with PD (64 male; aged 38–81, median 62 years), 18 patients with probable, possible, or pathologically proven MSA. Both types of MSA were included with predominant parkinsonism (MSA-P) and predominant cerebellar ataxia (MSA-C) (seven male; aged 51–80, median 64.5 years) and 31 age-matched controls (15 male; aged 43–77, median 61.5 years). Of the patient groups, 65 PD and 11 MSA patients were treated with levodopa and/or dopamine agonist; 54 PD and seven MSA patients were without dopaminergic treatment. In the PD group, 54 PD patients were treated for arterial hypertension; the others were without antihypertensive drugs. Disease duration in PD was 0.5–22 years, median 3.0; in MSA 1–7 years, median 2.0 years (Table 1).

### 3.2. PD Patients

**Figure 1:**
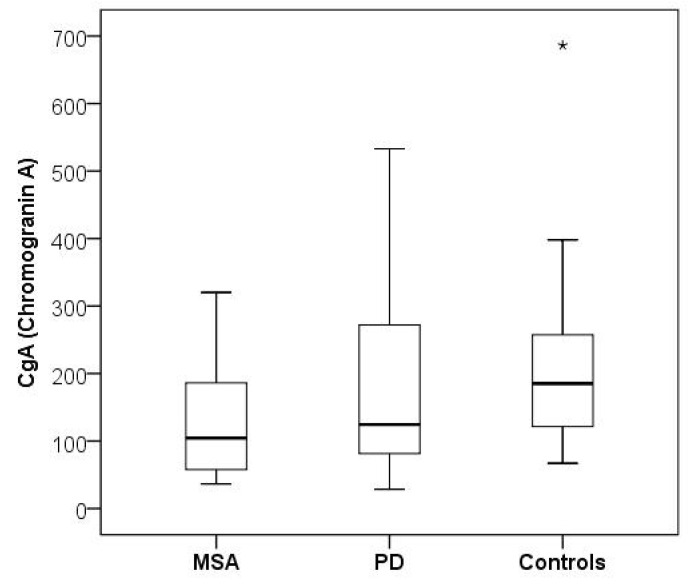
CSF CgA levels in multiple system atrophy, Parkinson’s disease, and control group. CSF, cerebrospinal fluid; CgA, chromogranin A; MSA, multiple system atrophy; PD, Parkinson’s disease. “*” means an isolated control case with CSF CgA level of 686.3 µg/L. The values of the other control patients were 67–398 µg/L.

**Figure 2:**
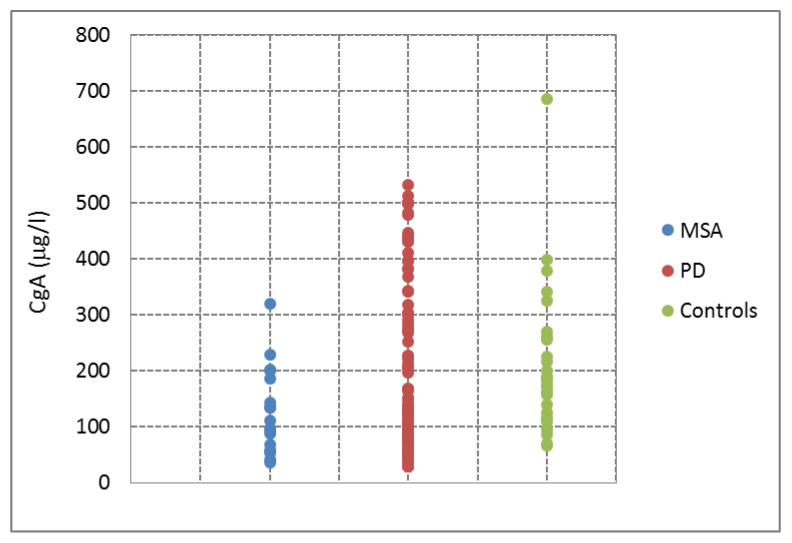
CSF CgA levels in CSF in multiple system atrophy, Parkinson´s disease, and control group. CSF, cerebrospinal fluid; CgA, chromogranin A; MSA, multiple system atrophy; PD, Parkinson´s disease.

There was no statistically significant difference in CSF CgA between the PD patients and the controls (median 124.5 vs. 185.2 µg/L; *p* = 0.057) (Figure 1 and Figure 2; Table 2). Absolute values of CgA in PD ranged from 28.6 to 532.9 µg/L, in the control group from 67.0 to 398 µg/L; there was an isolated control with 686.3 µg/L. Spearman’s correlation did not show a significant association between CSF CgA and age (correlation coefficient r = 0.13, *p* = 0.888), disease duration (minimum 0.5 years, maximum 22 years; correlation coefficient r = −0.027, *p* = 0.774), or levodopa dose equivalent (maximum 1945 mg/day; correlation coefficient r = −0.043, *p* = 0.641). There was no significant difference in CSF CgA in PD patients with arterial hypertension and PD patients without arterial hypertension (Table 3). There was no statistically significant difference in the age of PD patients compared to the control group (*p* = 0.886).

### 3.3. MSA Patients

CSF CgA levels were significantly lower in patients with MSA than in the control group (median 104.4 vs. 185.2; *p* = 0.014) (Figure 1 and Figure 2; Table 2). Absolute values of CgA in MSA ranged from 36.5 to 320.2 µg/L; CgA levels in the control group are mentioned above. There was no significant difference in CSF CgA between PD and MSA patients (*p* = 0.372). Spearman’s correlation did not show a significant association between CSF CgA and disease duration (correlation coefficient r = −0.074, *p* = 0.770) or levodopa dose equivalent (correlation coefficient r = −0.043, *p* = 0.641). There was no statistically significant difference in the age of MSA patients compared to the control group (*p* = 0.301).

## 4. Discussion

Our previous studies showed lower levels of CSF CgA in early stage PD [7,8]; lower levels of CSF CgA have also been found in advanced PD [10]. These studies were limited by small samples of patients. In the presented extended study, CSF CgA levels in PD patients tended to be lower than in the control group (median 124.5 vs. 185.2 µg/L); however, the results did not reach statistical significance. Similar results were recently published by Gmitterova et al. [6]. Lower CSF CgA levels in PD were also reported by Rotunno et al. [11]. We suggest that the lower levels of CgA are a consequence of the ongoing neurodegenerative process with a loss of catecholaminergic cells.

The reason for the non-significant result in our cohort may be the finding of strongly elevated CSF CgA in 13 patients (10.9%), who had CSF CgA levels above 400 µg/L, which was higher than the values of the control group. We do not have an explanation for this finding. None of these patients were diagnosed with other neurological or psychiatric disorders that could affect the CSF CgA level. Elevations in plasma CgA are principally associated with neuroendocrine tumor disease; increased levels may also occur in nongastrointestinal cancers, endocrine disease (such as pheochromocytoma or hyperthyroidism), gastrointestinal disorders, renal insufficiency, chronic inflammatory, and cardiovascular diseases [14]. In the group of PD patients with elevated CSF CgA, only six (46%) had arterial hypertension, the other patients had none from the abovementioned comorbidities; four (30%) patients were free of any comorbidity. None of the patients were diagnosed with pheochromocytoma or other tumor type. Disease duration was also not the reason, as it ranged from 0.5 to 22 years.

In our previous study, we found lower CSF CgA in PD patients with OH than in PD without OH [7]. This was presumed to be a consequence of the loss of brainstem catecholaminergic neurons resulting in impaired sympathetic reflex functions. In MSA patients, severe depletion of catecholaminergic neurons of the C1 and A1 areas in the ventrolateral medulla was pathologically proven; this finding is thought to contribute to OH, which is a prominent feature of MSA [15]. With regard to these findings, we measured CSF CgA in the MSA patients. CSF CgA levels were significantly lower than in the control group. Compared to PD, however, there was no significant difference in CSF CgA.

In both groups, PD and MSA, no significant association between CSF CgA and disease duration was observed. This suggests that central catecholaminergic cell depletion is present from the early stages of the disease, probably more severely in MSA than in PD.

Interestingly, there was not a case of strongly elevated CSF CgA levels in MSA as there was in PD. This may be due to the smaller number of MSA patients or by a different pathophysiology of these two diseases.

## 5. Conclusions

Chromogranin A and other peptides from the chromogranin-secretogranin family have been recently studied as potential biomarkers of PD. In our extended study, we observed a tendency toward lower CSF CgA levels in both PD and MSA patients compared to the control group, but the difference reached statistical significance only in MSA. In PD, statistical significance was not reached, probably because of strongly elevated CSF CgA in some patients, which we cannot explain. Taken together, CgA may have a potential as a biomarker in PD and MSA; however, further studies on larger numbers of patients are needed to draw conclusions.

## Figures and Tables

**Table 1:** Patients’ characteristics.

	Number	Gender (M/F)	Age (Years) Median (Min–Max)	Disease Duration (Years) Median (Min–Max)	Dopaminergic Treatment (with/without)
PD	119	64/55	62.0 (38–81)	3.0 (0.5–22)	65/54
MSA	18	7/11	64.5 (51–80)	2.0 (1–7)	11/7
Controls	31	15/16	61.5 (43–77)	-	-

PD: Parkinson’s disease; MSA: multiple system atrophy; M: male; F: female.

**Table 2:** CSF CgA levels in multiple system atrophy, Parkinson’s disease, and control group.

		MSA	PD	Control
CgA(µg/L)	Mean	125.3	184.8	207.4
	SD	76.7	141.6	124.2
	Median	104.4	124.5	185.2
	Minimum	36.5	28.6	67.0
	Maximum	320.2	532.9	686.3

CSF: cerebrospinal fluid; CgA: chromogranin A; MSA: multiple system atrophy; PD: Parkinson’s disease.

**Table 3:** CSF CgA levels in Parkinson’s disease patients with and without arterial hypertension.

		AH	Without AH	
CgA(µg/L)	Median	124.5	123.8	0.943
	Minimum	29	39	
	Maximum	500	533	

CSF: cerebrospinal fluid; CgA: chromogranin A; MSA: multiple system atrophy; PD: Parkinson’s disease; AH: arterial hypertension.

## Data Availability

The data that supports the findings of this study are available in Table 1, Table 2 and Table 3 as part of the article. More data are available from the corresponding author, upon reasonable request.

## References

[1] Taupenot L., Harper K.L., O’Connor D.T. (2003). The chromogranin-secretogranin family. N. Engl. J. Med..

[2] Nobels F.R.E., Kwekkeboom D.J., Bouillon R., Lamberts S.W.J. (1998). Chromogranin A: Its clinical value as marker of neuroendocrine tumours. Eur. J. Clin. Investig..

[3] Sahu B.S., Sonawane P.J., Mahapatra N.R. (2010). Chromogranin A: A novel susceptibility gene for essential hypertension. Cell. Mol. Life Sci..

[4] Pedrero-Prieto C.M., Garcia-Carpintero S., Frontinan-Rubio J., Llanos-Gonzalez E., Garcia C.A., Alcain F.J., Lindberg I., Duran-Prado M., Peinado J.R., Rabanal-Ruiz Y. (2020). A comprehensive systematic review of CSF proteins and peptides that define Alzheimer’s disease. Clin. Proteom..

[5] Kaiserova M., Grambalova Z., Otruba P., Stejskal D., Vranova H.P., Mares J., Mensikova K., Kanovsky P. (2017). Cerebrospinal fluid levels of chromogranin A and phosphorylated neurofilament heavy chain are elevated in amyotrophic lateral sclerosis. Acta Neurol Scand..

[6] Gmitterova K., Varges D., Schmitz M., Zafar S., Maass F., Lingor P., Zerr I. (2020). Chromogranin A Analysis in the Differential Diagnosis Across Lewy Body Disorders. J. Alzheimers Dis..

[7] Kaiserova M., Vranova H.P., Galuszka J., Stejskal D., Mensikova K., Zapletalova J., Mares J., Kanovsky P. (2015). Orthostatic hypotension is associated with decreased cerebrospinal fluid levels of chromogranin A in early stage of Parkinson disease. Clin. Auton. Res..

[8] Kaiserova M., Vranova H.P., Stejskal D., Mensikova K., Kanovsky P. (2013). Cerebrospinal fluid levels of chromogranin A in the treatment-naïve early stage Parkinson’s disease: A pilot study. J. Neural Transm..

[9] Xu D.J., Wei L.Y., Li H.F., Zhang W.Q. (2019). Serum levels of chromogranins and secretogranins correlate with the progress and severity of Parkinson’s disease. Kaohsiung J. Med. Sci..

[10] Oconnor D.T., Cervenka J.H., Stone R.A., Parmer R.J., Francobourland R.E., Madrazo I., Langlais P.J. (1993). Chromogranin-a Immunoreactivity in Human Cerebrospinal-Fluid-Properties, Relationship to Noradrenergic Neuronal-Activity, and Variation in Neurologic Disease. Neuroscience.

[11] Rotunno M.S., Lane M., Zhang W.F., Wolf P., Oliva P., Viel C., Wills A.M., Alcalay R.N., Scherzer C.R., Shihabuddin L.S. (2020). Cerebrospinal fluid proteomics implicates the granin family in Parkinson’s disease. Sci. Rep..

[12] Gibb W.R., Lees A.J. (1988). The relevance of the Lewy body to the pathogenesis of idiopathic Parkinson’s disease. J. Neurol. Neurosurg. Psychiatry.

[13] Gilman S., Wenning G., Low P., Brooks D., Mathias C., Trojanowski J., Wood N.W., Colosimo C., Durr A., Fowler C. (2008). Second consensus statement on the diagnosis of multiple system atrophy. Neurology.

[14] Modlin I.M., Gustafsson B.I., Steven F Moss S.M., Pavel M., Tsolakis A.V., Kidd M. (2010). Chromogranin A—Biological function and clinical utility in neuro endocrine tumor disease. Ann. Surg. Oncol..

[15] Benarroch E.E. (2003). Brainstem in multiple system atrophy: Clinicopathological correlations. Cell Mol. Neurobiol..

